# Comparison of HIV characteristics across 3 datasets: the Korea HIV/AIDS Cohort Study prospective, retrospective, and national reporting system

**DOI:** 10.4178/epih.e2024055

**Published:** 2024-06-18

**Authors:** Yunsu Choi, Jun Yong Choi, Bo Youl Choi, Bo Young Park, Shin-Woo Kim, Joon Young Song, Jung Ho Kim, Sang Il Kim

**Affiliations:** 1 Department of Preventive Medicine, Hanyang University College of Medicine, Seoul, Korea; 2 Institute for Health and Society, Hanyang University, Seoul, Korea; 3 Department of Internal Medicine, AIDS Research Institute, Yonsei University College of Medicine, Seoul, Korea; 4 Department of Internal Medicine, Kyungpook National University School of Medicine, Daegu, Korea; 5 Department of Internal Medicine, Korea University College of Medicine, Seoul, Korea; 6 Division of Infectious Disease, Department of Internal Medicine, Seoul St. Mary’s Hospital, College of Medicine, The Catholic University of Korea, Seoul, Korea

**Keywords:** Cohort studies, Selection bias, Research design, HIV, Acquired immunodeficiency syndrome

## Abstract

**OBJECTIVES:**

The Korea HIV/AIDS Cohort Study has been conducted prospectively for 18 years. However, it faces limitations in representing the entire population of patients with human immunodeficiency virus (HIV) in Korea. To address these limitations and validate the study design, we analyzed characteristics across several HIV datasets.

**METHODS:**

We compared epidemiological and clinical characteristics from 3 datasets: the Korea HIV/AIDS Cohort Study (dataset 1, n=1,562), retrospective cohort data (dataset 2, n=2,665), and the national HIV reporting system of the Korea Disease Control and Prevention Agency (KDCA) (dataset 3, n=17,403).

**RESULTS:**

The demographic characteristics of age, sex, and age at HIV diagnosis did not differ significantly across datasets. However, dataset 3 contained a higher proportion of patients diagnosed after 2008 (69.5%) than the other datasets. Regarding transmission routes, same-sex contact accounted for a greater proportion of dataset 1 (59.8%) compared to datasets 2 (20.9%) and 3 (32.6%). The proportion of patients with CD4 T-cell counts below 200/mm^3^ at HIV diagnosis was higher in datasets 1 (39.4%) and 2 (33.3%) compared to dataset 3 (16.3%). Initial HIV viral load measurements were not obtained for dataset 3.

**CONCLUSIONS:**

The Korea HIV/AIDS Cohort Study demonstrated representativeness regarding the demographic characteristics of Korean patients. Of the sources, dataset 1 contained the most data on transmission routes. While the KDCA data encompassed all HIV patients, it lacked detailed clinical information. To improve the representativeness of the Korea HIV/AIDS Cohort Study, we propose expanding and revising the cohort design and enrolling more patients who have been recently diagnosed.

## GRAPHICAL ABSTRACT

[Fig f1-epih-46-e2024055]

## Key Message

• The research data collected from the cohort exhibited bias relative to nationally reported data.

• We propose 2 types of cohort enrollment—an interval cohort and a clinical cohort—that reflect the characteristics of infected individuals through reorganization of the cohort design.

• Within the national insurance system, a customized big data platform (insurance cohort) should be established to monitor the medical claims of individuals who have visited healthcare facilities outside of the participating institutions.

• In response to the inherent limitations of cohort and claims data, a combined dataset should be created to increase the utility of the research.

## INTRODUCTION

The Korea HIV/AIDS Cohort Study (KoCosHIV) has been underway since December 2006, with funding from the Korea Disease Control and Prevention Agency (KDCA) [1,2]. A total of 1,593 patients with human immunodeficiency virus (HIV) were enrolled, 943 of whom underwent follow-up until September 2022. The KoCosHIV was structured as an ambidirectional cohort study, examining data retrospectively from the time of diagnosis to study enrollment and then prospectively at 6-month intervals.

In accordance with our research ethics regulations, which are based on the Bioethics Act, only adults aged 18 years and older who voluntarily consented were eligible to participate in the study. Consequently, we could not include all individuals with HIV who visited the participating institutions. The subgroup under investigation (individuals infected with HIV) may have been particularly interested in their health, as indicated by both a high rate of study participation and a high retention rate for follow-up after enrollment [3]. Additionally, many patients experienced a favorable clinical course or did not show disease progression. Restricting enrollment to patients aged over 18 years may impact the reliability of the long-term follow-up cohort study. This demographic may exhibit a comparatively high personal interest in health issues and a cooperative attitude toward medical care, potentially yielding better outcomes [4,5].

To strengthen the representativeness of the KoCosHIV, we compared the clinical and epidemiological characteristics of patients with HIV who were not enrolled in the KoCosHIV.

In patient research, research tools must be periodically reorganized to capture changing clinical and epidemiological characteristics or treatment patterns in real time. The study design should also be restructured to minimize interventions. Particularly, the need arises for systematic review of long-term studies, as cohort enrollees tend to exhibit better adherence to treatment than those not enrolled [6]. Therefore, we compared the characteristics of the study participants with those of non-participants to identify any differences and to validate the current study design.

## MATERIALS AND METHODS

### Participants

Three raw data sources were used in this study. The summary characteristics of each dataset are as follows.

#### The KoCosHIV (cohort enrollees; dataset 1)

Data were collected from December 2006 to December 2020 across 21 hospitals, with 1,562 participants enrolled (cohort enrollees) [1]. Dataset 1 included: (1) age, sex, age at diagnosis, year of diagnosis, initial CD4 T-cell count, initial HIV viral load, accompanying diseases, treatment-related events, medications, clinical outcomes and comorbidities, and the natural course of the disease as recorded in medical records; (2) responses to questionnaires that investigated HIV transmission route and quality of life, obtained through direct interviews; and (3) 16-mL blood samples collected during each visit for further examination. The study was conducted as an open cohort from 2006 to 2022, allowing for the voluntary participation of individuals infected with HIV at any time.

#### The Korea HIV/AIDS Retrospective Cohort Study (dataset 2)

Data were obtained from patients who visited a participating institution between November 2015 and August 2016. These patients had medical records indicating an HIV diagnosis but did not participate in the KoCosHIV (non-cohort patients) [7]. Dataset 2 included 2,663 patients and consisted of a mandatory record-based survey (dataset 1). The primary purpose of dataset 2 was to identify potential selection bias within the KoCosHIV. It contained information on age, sex, age at diagnosis, year of diagnosis, initial CD4 T-cell count, initial HIV viral load, accompanying disease, treatment-related events, medications, clinical outcomes and comorbidities, and the natural course of the disease, based solely on patient medical records. Data duplication can occur whenever individuals visit multiple institutions, with particular risk in this case if the institutions are part of the KoCosHIV study. Additionally, individuals who did not participate in the KoCosHIV during the survey period for dataset 2 may have subsequently enrolled and thus would be included in dataset 1. As a result, datasets 1 and 2 cannot be considered entirely independent.

#### 2021 KDCA HIV epidemiological report data (surveillance; dataset 3)

These data were utilized to assess potential differences in characteristics between the cohort participants and all individuals with HIV in Korea. However, the study only included Korean citizens over the age of 18, thereby excluding foreign-born residents living with HIV/AIDS from the reported data [8]. Dataset 3 consisted of frequency data extracted from KDCA surveillance records, which included age, sex, age at diagnosis, year of diagnosis, initial CD4 T-cell count, initial HIV viral load, and presumed transmission route as reported by patients on self-administered questionnaires. This dataset differed from datasets 1 and 2 in that it did not rely on individually collected data. Instead, it compiled relevant indicators from existing reports as descriptive statistics. Due to the nature of HIV surveillance data, which in Korea are based on self-reporting, dataset 3 also contained information on patients who were included in datasets 1 and 2. The KDCA data were encrypted in accordance with the Personal Information Protection Act, making it infeasible to separate them from the study dataset or to integrate them.

### Statistical analysis

Existing and retrospective cohort data are not independent and may overlap. Although the reported data included participants from cohort studies and duplicate participants, no matching variables were available. Consequently, we were unable to conduct statistical tests due to the inability to construct integrated datasets. Therefore, all 3 datasets were treated as independent, and their distributions were compared by frequency. Our analysis accounted for all missing values, inherently indicating the limited information available about the study population. All analyses were conducted using SAS Enterprise Guide 7.1 (SAS Institute Inc., Cary, NC, USA).

### Ethics statement

The study received approval from our Institutional Bioethics Committee (approval Nos. #CTSC-IRBF-18-1, #4-2022-0495, and #4-2022-0676). Furthermore, we utilized data exclusively from participants who provided informed consent for involvement. This consent was obtained after an explanation of the study during the recruitment phase. No additional samples or materials were collected specifically for this research.

## RESULTS

### Comparison of characteristics by data source

#### Demographic characteristics

The numbers of participants in the 3 datasets were 1,562, 2,665, and 17,403 (Table 1). Approximately 93% of the patients were male, and 50% to 55% were young adults in their 20s or 30s. In the cohort data (datasets 1 and 2), the largest proportion of patients were in their 30s. However, the reported data (dataset 3) contained a higher proportion of patients in their 20s. In dataset 1, the number of enrolled patients who were diagnosed before 1996 was smaller than the actual number of HIV/AIDS cases in Korea (dataset 3) for the same period. The proportion of patients diagnosed between 1997 and 2007 was greater than that in dataset 3. Additionally, the proportion of patients diagnosed after 2008 in dataset 1 was lower than in dataset 3.

#### Transmission route

Data were collected using various methods: questionnaires completed by the study team during direct interviews for dataset 1, review of medical records for dataset 2, and self-administered questionnaires completed by patients from the KDCA surveillance records for dataset 3. The percentage of cases attributed to same-sex contact was higher in dataset 1 (59.8%) compared to dataset 2 (20.9%) and dataset 3 (32.6%) (Table 1). The proportion of opposite-sex contact was greater in dataset 3 (43.0%) than in datasets 1 (33.9%) and 2 (15.8%). Intravenous drug use and vertical transmission were reported at rates of less than 0.1% for all datasets. Dataset 3 contained numerous missing values for relevant questions, resulting in a higher percentage of cases with an unknown transmission route (24.1%).

#### HIV status at diagnosis

The data lacked initial CD4+ cell counts for more than 50% of the cases reported by the KDCA, as well as for approximately 1-20% of the cohort data. Nevertheless, the proportion of individuals with serological acquired immune deficiency syndrome (AIDS), indicative of a delayed diagnosis, ranged from 16.3% to 39.4% across all data sources.

At diagnosis, the proportion of patients with HIV RNA copy numbers of ≥ 100,000 (copies/mL) was 30.9% in dataset 1 and 20.8% in dataset 2. Dataset 3 contained no records of HIV viral load. In dataset 2, the HIV viral load at diagnosis was unknown for 34.1% of cases (Table 1).

### Treatment-related characteristics after diagnosis: comparison of cohort data

Treatment-related clinical data were analyzed by extracting records of treated participants from both cohort datasets (1 and 2; Table 2). No relevant data were available from dataset 3. Over the entire follow-up period, the proportion of participants with nadir CD4 T-cell counts of less than 200/mm^3^ was 41.6% in dataset 1 and 37.1% in dataset 2. The proportion of participants with a peak HIV viral load exceeding 100,000 copies/mL of blood was 35.6% in dataset 1 and 27.5% in dataset 2.

Regarding age at the start of treatment, both datasets exhibited a similar age distribution, with the most common ages being in the 30s, followed by the 40s and then the 20s. Most enrollees began antiretroviral therapy (ART) after 2008 in both dataset 1 and dataset 2, with rates of 66.3% and 59.4% respectively. The most common initial ART regimen consisted of 2 nucleoside reverse transcriptase inhibitors (NRTIs) with a protease inhibitor (PI), which was administered to 26.7% of patients in dataset 1 and 24.1% in dataset 2. The next most common was a regimen of 2 NRTIs with non-nucleoside reverse transcriptase inhibitors (NNRTIs), followed by a protocol of 2 NRTIs with an integrase inhibitor. Most patients initiated ART at the time of HIV diagnosis (95.7% in dataset 1 and 94.2% in dataset 2), while a minority delayed starting ART (4.3% in dataset 1 and 5.8% in dataset 2). During the followup periods, 55.5% of patients in dataset 1 and 46.6% in dataset 2 changed their ART regimen 2 times to 5 times for various reasons, including medication side effects, the availability of newer drugs requiring fewer pills, resistance, and the desire for a more convenient pill-taking regimen (Supplementary Material 1). Recovery of CD4 T-cell count (defined as an increase of at least 100 cells/mm^3^ at 48 weeks after ART initiation) was achieved by 74.2% of patients in dataset 1 and 55.7% in dataset 2, although dataset 2 had more missing values. Viral suppression—defined as less than 50 copies/mL of blood at 32 weeks after initial ART—was achieved by 59.3% of patients in dataset 1 and 58.4% in dataset 2. The initial treatment regimen was NNRTIs with PI. Among participants experiencing ART, 95.7% in dataset 1 and 94.2% in dataset 2 received highly active antiretroviral therapy at the time of initial treatment. Of the non-enrolled patients, 83.5% underwent fewer than 5 times ART changes; in contrast, 73.2% of the enrolled cohort experienced fewer than 5 times ART changes (Table 2).

The questionnaire addressing changes in ART regimen and reasons for discontinuation was exclusively available to cohort participants (dataset 1). Side effects were the predominant cause for changes, accounting for approximately 30%, followed by prescription modifications due to the introduction of new drugs or combinations (about 19.4%). Other frequent reasons included treatment failure (6.2%), drug interactions (5.0%), and drug resistance (3.9%). Notably, 22.0% of participants were unsure of the rationale behind changes to or discontinuation of their medication (Supplementary Material 1).

## DISCUSSION

### Synthesis and summary

This study was performed to understand the biases introduced by the design of an existing cohort study and to revise that design accordingly. Objective comparisons of metrics necessitate statistical validation of comparable measurements, which typically involves excluding missing values; however, this preliminary datacleaning process was not conducted systematically in our study. Unlike other cohort studies, our primary objective was to address selection bias from the outset, ensuring that our cohort of HIV cases reflected the characteristics of people living with HIV (PLHIV) in Korea. Obtaining voluntary consent from PLHIV to participate in research poses considerable challenges in real-world clinical practice. PLHIV often exhibit reluctance to engage in research due to concerns regarding stigma and feelings of being monitored. Consequently, despite being structured as an open cohort, the KoCosHIV has seen a dearth of new enrollments since the inception of the early cohort study period for dataset 1 (2006-2008). Additionally, the follow-up rates of participants who did enroll remain low, at approximately 60%. Moreover, missing values were not excluded from comparable metrics in our analysis, as we consider them valuable metrics for illustrating this phenomenon. Although the presence of missing values can influence outcomes, they also highlight the inherent constraints in obtaining information about individuals based on the characteristics of the study population.

During the cohort study for dataset 1, we carefully evaluated the rate of missing data for each question, using this information to refine the survey instrument. To minimize biases in the construction of the cohort, we redesigned our research approach to incorporate a variety of data sources. This step is crucial because relying on cohorts of relatively healthy individuals with HIV may not provide a full picture of the characteristics of all infected individuals at the national level. Furthermore, due to the multi-institutional nature of our study, we have provided systematic standardization training for investigators, such as doctors and nurses, to reduce potential biases introduced by the use of multiple data collectors during the data collection phase. We have also introduced online surveys accessible via personal devices like smartphones or tablet computers, which encourages participation from individuals who may be reluctant to join the study due to concerns about exposure during data collection. By utilizing medical claims records, we can determine whether participants have received medical care at facilities other than the participating institutions. This allows for a thorough comparison of records for individuals who are receiving HIV-related care or prescription treatments from non-participating facilities. Consequently, the revised research design can function as the definitive dataset, facilitating diverse comparisons of characteristics among infected individuals.

Among the cohort enrollees, the most common time from HIV diagnosis to initial treatment was 2 years or more (39.1% of the cohort), followed by a period of 3 months or less (33.9%). In contrast, 61.1% of non-enrollees initiated treatment within 3 months of diagnosis. Dataset 1 included patients diagnosed before and during the early 2000s, a time when guidelines recommended an individualized approach to ART initiation for asymptomatic patients with CD4+ T cell counts above 200 cells/mm^3^ [9]. This pattern was echoed in the time from diagnosis to death and from the start of treatment to death (Supplementary Material 2). Cross-tabulation to assess synchrony between relevant indicators revealed that cohort enrollees had the highest proportion of long-term survivors, with periods of more than 2 years from diagnosis to treatment and from treatment initiation to death (Supplementary Material 3). Non-enrollees had the highest proportions of individuals whose periods from HIV diagnosis to initial treatment and from treatment to death occurred within 3 months, suggesting that disease characteristics, such as severity, differed markedly based on cohort enrollment status.

Although we could not determine all causes of death, few cohort enrollees died from AIDS or AIDS-related diseases. In contrast, the non-enrollee cohort experienced the highest number of deaths directly attributable to AIDS (Supplementary Material 4). Across all datasets, a clearer delineation of antecedent and immediate causes of death is needed. Most medical records in datasets 1 and 2 documented the medical condition preceding death, except in cases involving loss to follow-up. However, dataset 3, consisting solely of KDCA reports, only indicated overall status (survival or death). The representativeness of the cohort could be increased by improving the detail of data collection for KDCA reports, within the bounds of the Personal Information Protection Act, and by providing standardized training for the cohort study team. Furthermore, we were unable to examine certain characteristics because the KDCA data lacked surveys on the timing of diagnosis, treatment, and death. Nonetheless, a clear difference was observed based on whether participants who visited the same institution were enrolled in the cohort.

### Limitations of previous study designs

A clear selection bias was observed in these patient cohort studies with long-term follow-up periods. Specifically, patients with severe clinical conditions at enrollment were often excluded from the study, resulting in the inclusion of only relatively healthy patients. Additionally, the percentages of patients diagnosed after 2008 were considerably lower in the cohort groups. Despite the inherent requirement of these cohort studies to be conducted over extended periods and to capture the longitudinal nature of the disease, it is essential to enroll newer patients to reflect the current characteristics of the condition.

We acknowledge that the KoCosHIV, as conducted over the past 15 years, presents a biased representation of participant characteristics; additionally, enrolling newly infected individuals is challenging due to social stigma. Consequently, informed by the outcomes of additional research, we opted to revise and improve the study design, recognizing that these factors could influence policy development.

### Rationale for cohort design revisions

Cohort studies track a population over time, often identifying potentially at-risk groups at the beginning of the study, while also evaluating exposure to risk factors. Exposure can be characterized in various ways, such as the occurrence or exacerbation of a disease, with risk typically assessed through statistical methods. Cohort studies are commonly categorized into prospective and retrospective studies, distinguished by the timing with which exposure is evaluated [10]. Prospective studies monitor participants over time, gathering data as their characteristics evolve. In contrast, retrospective studies collect past data from participants. A retrospective study enrolls participants after an outcome has been determined, and then assesses exposures from the baseline to before the outcome occurred or before the study began. Retrospective studies are prone to recall bias during the survey process, which is why findings from prospective studies are often given more weight than those from retrospective studies [11]. Researchers usually classify a study as prospective if it involves a population-based cohort with follow-up [12]. However, the designation of the cohort at the time of investigation does not fully capture the study’s nature. This is because some cohorts are revisited at specific intervals, while studies may also utilize data from medical records or healthcare claims not originally collected for research purposes.

An interval cohort study involves repeated surveys administered at specific intervals, such as every 6 months or annually, using standardized survey instruments to explore research topics beyond medical treatment. However, voluntary participation can introduce selection bias based on participant characteristics, and self-reported treatment histories are susceptible to recall bias. Furthermore, the necessity for participants to complete surveys repeatedly may exacerbate selection bias [13].

A clinical cohort study is based on medical records, with research questions posed separately following a medical visit. This approach can reflect actual medical records and provide precise details regarding prescription history. However, reliance on medical records can lead to medical surveillance bias, potentially inflating the true risk of disease and resulting in disparities in the quality of information recorded by different clinicians. Furthermore, because this approach relies on medical records, it carries a risk of missing data from individuals who do not seek care from a participating healthcare provider [13].

In Korea, a health insurance cohort consists of records from medical insurance claims submitted by subscribers to the Korean National Health Insurance System (NHIS). Every Korean citizen or legal resident is entitled to coverage through the NHIS or medical benefits. When a subscriber seeks care at a medical institution, the Health Insurance Review and Assessment Agency (HIRA) reviews the submitted medical bills. HIRA then reports the review outcomes to the NHIS, which settles the charges with the medical institution based on these findings [14]. The Korean NHIS database includes information on a variety of research topics and medical institutions, as well as data on procedures, tests, prescriptions, diagnoses, and health insurance eligibility. However, it lacks test results, which precluded the analysis of clinical courses in the present study. The database enables analyses of a national population sample, potentially uncovering rare diseases or unexpected drug side effects. Nevertheless, since the data are not collected specifically for research purposes, a systematic discussion of operational definitions is necessary. Moreover, because the claims data include only services eligible for insurance reimbursement, it is impossible to ascertain what is excluded. Additionally, the absence of outcome information on behaviors, tests, prescriptions, and diagnoses—like what is found in medical records—may lead to an overestimation of actual cases. To reduce bias, periodic updates from sources based on the 3 cohort designs are beneficial, as each can compensate for the limitations of the others.

### Revised cohort study design

To revise the cohort study design, we devised 2 separate enrollment options: an interval cohort and a clinical cohort. The primary inclusion criteria encompassed those who consented to participate in the study, had been infected with HIV, and were over 18 years old. The interval cohort involved the collection of approximately 16 mL of blood and the administration of a self-reported questionnaire under researcher supervision, which included psychosocial factors. The criteria for registration in these databases were as follows: (1) individuals who had received an HIV diagnosis within the past year, (2) young adults in their 20s and 30s at the time of diagnosis, and (3) those who did not meet the above conditions but exhibited high blood viral loads. Registration was also possible for patients who wished to enroll or whose attending physician deemed sample collection necessary, even if the criteria were not met. In contrast, participants in the clinical cohort did not provide blood samples. Instead, they completed self-report questionnaires regarding their disease history, based primarily on their medical records and excluding socio-psychological factors. The enrollment criteria for this cohort gave priority to recently diagnosed individuals who were not included or enrolled according to the interval cohort criteria. Although the registration forms differed, the survey questionnaire format was the same, allowing for the integration and subsequent use of the 2 datasets. This design method is ethical, as it grants research participants the autonomy to choose their type of research involvement. From the researcher’s perspective, it offers the key advantage of securing a diverse participant pool. The 2 sets of information directly correspond to the primary data, facilitating the real-time identification of infected individuals in a clinical setting. In this study, the number of participants available for electronic medical record review was determined after the initiation of HIV medical services at the 16 participating institutions. Excluding 3 institutions that did not respond, data on 8,267 individuals with HIV were available from the remaining 13 institutions. Of these, 6,285 (76.0%) had accessed HIV-related services in the previous year (as of January 1, 2021). Among them, 660 (approximately 10.5% of all ongoing patients) were newly diagnosed after January 1, 2020, with 442 (66.7%) in their 20s and 30s.

We aimed to enroll roughly 200 new interval cohort participants each year, with the goal of achieving a total of 1,500 registrations within 3 years. Additionally, to mitigate participant attrition due to severe disease, we sought to recruit around 1,000 clinical cohort participants annually.

Furthermore, utilizing the NHIS claims big data, which encompasses approximately 98% of the nation’s population, we constructed a customized insurance cohort. This cohort compares roughly 98% of the claims data for infected individuals against domestic report data. While these data do not support the verification of clinical outcomes and confirmed cases, they offer the benefit of monitoring disease-related trends among infected individuals in Korea through medical claims information.

Traditionally, data from different sources could not be combined due to a lack of sufficient information for integration. With the revised KoCosHIV data, instead of allowing participants to change their participation status at any time, we assign them a traceable and encrypted ID, enabling independent management. When combining interval and clinical cohort data with claims data, we employ a pseudonymization technique. This method generates a combination key using a set of attributes—sex, age group, healthcare provider visited, and date of the visit—to merge the data without including personally identifiable information, such as social security numbers. If no information is missing, matching rate is high, exceeding 90%. We integrated cohort and claims data to address the limitations inherent in cohort data, which may lack records due to challenges in tracking visits to different institutions, and in claims data, which are typically missing information on clinical outcomes. The refined and combined dataset included approximately 1,446 participants who consented to the creation of these integrated data.

Through the revision of research design, we can establish clear criteria for target selection and utilize various data sources to increase research applicability, including the generation of findings on a range of topics. The institutional review board approved the revised designs for each research institution in August 2022, and investigations have been ongoing since October 2022. As per the KoCosHIV data distribution standard, data collected within the same timeframe can be prepared for distribution if a refinement rate of 90% or higher is achieved on at least 2 occasions during the data cleaning process. The data from the revised design are slated for distribution starting in 2024. To use the cohort data, separate internal reviews are necessary, and external researchers, other than those part of the internal cohort team, may face restrictions regarding access to the data.

In conclusion, this study clarified the limitations of and discrepancies between pre-existing cohort studies and the available real-world data. Each dataset exhibited structural limitations and issues with the data collection system. To improve the representativeness of the KoCosHIV, substantial revisions to the study design and an expansion of data sources are required.

## Figures and Tables

**Figure f1-epih-46-e2024055:**
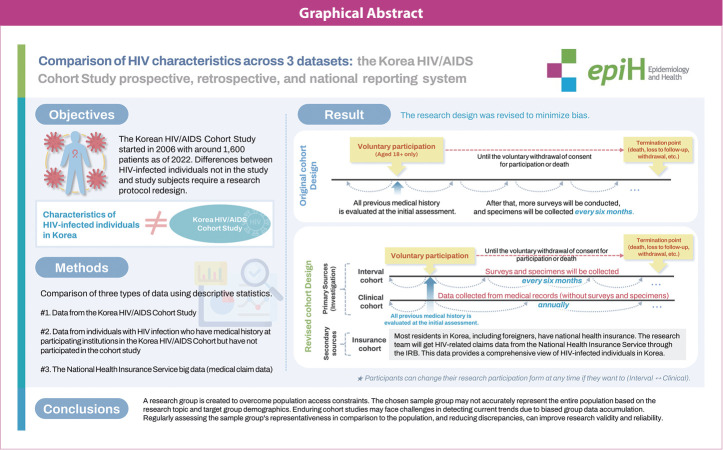


**Table 1.** General characteristics of HIV diagnosis

Characteristics	Dataset 1 (n=1,562)	Dataset 2 (n=2,665)	Dataset 3 (n=17,403)
Sex			
Male	1,460 (93.5)	2,479 (93.0)	16,250 (93.4)
Female	101 (6.4)	185 (6.9)	1,153 (6.6)
Missing	1 (0.1)	1 (0.1)	-
Age at diagnosis (yr)			
<20^1^	54 (3.5)	74 (2.8)	531 (3.1)
20-29	370 (23.7)	672 (25.2)	4,860 (27.9)
30-39	435 (27.8)	743 (27.9)	4,640 (26.7)
40-49	338 (21.6)	615 (23.1)	3,606 (20.7)
50-59	201 (12.9)	302 (11.3)	2,431 (14.0)
60-69	84 (5.4)	115 (4.3)	992 (5.7)
≥70	17 (1.1)	36 (1.3)	343 (2.0)
Unknown	63 (4.0)	108 (4.1)	-
Year of diagnosis			
≤1996	38 (2.4)	109 (4.1)	621 (3.6)
1997-2007	751 (45.7)	1,018 (38.2)	4,695 (27.0)
≥2008	804 (51.5)	1,431 (53.7)	12,087 (69.5)
Unknown	7 (0.4)	107 (4.0)	-
Transmission route			
Same-sex contact	934 (59.8)	557 (20.9)	5,666 (32.6)
Opposite-sex contact	530 (33.9)	422 (15.8)	7,481 (43.0)
IDU	1 (0.1)	1 (0.1)	9 (0.1)
Vertical transmission	0 (0.0)	1 (0.1)	9 (0.1)
Others^2^	95 (6.1)	1,542 (57.8)	46 (0.3)
Unknown	2 (0.1)	142 (5.3)	4,192 (24.1)
Initial CD4 cell count (cells/mm^3^)			
<200	616 (39.4)	888 (33.3)	2,843 (16.3)
200-349	424 (27.1)	557 (20.9)	1,884 (10.8)
350-499	279 (17.9)	358 (13.4)	1,332 (7.7)
≥500	234 (15.0)	322 (12.1)	1,211 (7.0)
Unknown	9 (0.6)	540 (20.3)	10,113 (58.2)
Initial HIV viral load (copies/mL)			
<10,000	471 (30.1)	517 (19.4)	-
10,000-54,999	411 (26.3)	460 (17.3)	-
55,000-99,999	183 (11.7)	224 (8.4)	-
≥100,000	482 (30.9)	554 (20.8)	-
Unknown	15 (1.0)	910 (34.1)	-

HIV, human immunodeficiency virus; IDU, injection drug use.

1 Dataset 1 included only adults aged >18 years, while datasets 2 and 3 included infants and teenagers.

2 Includes blood transfusion and blood coagulation.

**Table 2.** Comparison of ART-related clinical status by cohort enrollment

Variables	Dataset 1	Dataset 2
Total (n)	1,454	2,170
Nadir CD4 T-cell counts (cells/mm^3^)		
<200	605 (41.6)	805 (37.1)
200-349	444 (30.5)	508 (23.4)
350-499	131 (9.0)	219 (10.1)
≥500	49 (3.4)	78 (3.6)
Missing^1^	225 (15.5)	560 (25.8)
Peak HIV viral load (copies/mL)		
<10,000	165 (11.3)	228 (10.5)
10,000-54,999	330 (22.7)	475 (21.9)
55,000-99,999	183 (12.6)	232 (10.7)
≥100,000	517 (35.6)	596 (27.5)
Missing^1^	259 (17.8)	639 (29.4)
Age at initial ART (yr)		
<20^2^	22 (1.5)	28 (1.3)
20-29	307 (21.1)	408 (18.8)
30-39	419 (28.8)	632 (29.1)
40-49	383 (26.4)	524 (24.2)
50-59	208 (14.3)	269 (12.4)
60-69	95 (6.5)	107 (4.9)
≥70	17 (1.2)	27 (1.2)
Missing^1^	3 (0.2)	175 (8.1)
Year of ART initiation		
≤1996	6 (0.4)	11 (0.5)
1997-2007	483 (33.2)	696 (32.1)
≥2008	963 (66.3)	1,289 (59.4)
Missing^1^	2 (0.1)	174 (8.0)
Initial ART regimen		
2NRTI+PI	388 (26.7)	522 (24.1)
2NRTI+NNRTI	236 (16.2)	263 (12.1)
2NRTI+INI	138 (9.5)	238 (11.0)
Others	627 (43.1)	1,111 (51.2)
Missing^1^	65 (4.5)	36 (1.6)
Initial HAART		
Yes	1,391 (95.7)	2,044 (94.2)
No	63 (4.3)	126 (5.8)
No. of changes to ART line		
1	258 (17.7)	801 (36.9)
2-5	807 (55.5)	1,011 (46.6)
≥5	388 (26.7)	355 (16.4)
Missing^1^	1 (0.1)	3 (0.1)
CD4 recovery		
Yes	1,078 (74.2)	1,209 (55.7)
No	143 (9.8)	381 (17.6)
Missing^1^	233 (16.0)	580 (26.7)
Viral suppression		
Yes	862 (59.3)	1,268 (58.4)
No	576 (39.6)	714 (32.9)
Missing^1^	16 (1.1)	188 (8.7)

Values are presented as number (%). ART, antiretroviral therapy; HIV, human immunodeficiency virus; HAART, highly active antiretroviral therapy; NRTI, nucleotide reverse transcriptase inhibitor; NNRTI, non-nucleotide reverse transcriptase inhibitor; PI, protease inhibitor; INI, integrase inhibitor.

1 These participants had no reported test results over the entire study period.

2 Dataset 1 included only adults aged >18 years, while datasets 2 and 3 included infants and teenagers.

## Supplementary materials

Supplementary Material 1. Causes of changing/discontinuing medication until 2017

Supplementary Material 2. Duration of HIV diagnosis, initial ART, and death in the cohort data until 2017

Supplementary Material 3. Distribution of patients (n=number) according to intervals between diagnosis, starting ART, and all cause of death

Supplementary Material 4. Causes of death by cohort enrollment until 2017
