# Supplementary material for: Comparison of HIV characteristics across 3 datasets: the Korea HIV/AIDS Cohort Study prospective, retrospective, and national reporting system
Source: Epidemiol Health. 2024 Jun 18;46:e2024055. doi: 10.4178/epih.e2024055 (PMC11573489; doi:10.4178/epih.e2024055)
Supplement: Supplementary Material 1. — Causes of changing/discontinuing medication until 2017 [file epih-46-e2024055-Supplementary-1.docx]

Supplementary Materials 1. Causes of changing/discontinuing medication until 2017

| Cause of changing medication | | | Dataset 1^*^ |
| --- | --- | --- | --- |
|  | Side effects | | 1,336(29.8) |
|  | Changing to a combination drug | | 869(19.4) |
|  | Treatment failure | | 279(6.2) |
|  | Self-discontinuation (patients) | | 245(5.5) |
|  | Drug interaction | | 225(5.0) |
|  | Adding and changing drug | | 184(4.1) |
|  | Drug resistance | | 175(3.9) |
|  | Changing doses | | 141(3.1) |
|  | The convenience of taking medication (patient demand) | | 140(3.1) |
|  | Poor adherence | | 136(3.0) |
|  | Others | |  |
|  |  | Identify and prevent side effects | 86(1.9) |
|  |  | Drug supply issues (discontinued) and clinical trial participation | 84(1.9) |
|  |  | Pregnancy/Delivery | 13(0.3) |
|  |  | Prescription errors | 13(0.3) |
|  |  | Change back to a past combination | 7(0.2) |
|  | Unknown | | 987(22.0) |

^*^ Allows duplicate responses; this was not investigated in Datasets 2 and 3.
