# Supplementary material for: Comparison of HIV characteristics across 3 datasets: the Korea HIV/AIDS Cohort Study prospective, retrospective, and national reporting system
Source: Epidemiol Health. 2024 Jun 18;46:e2024055. doi: 10.4178/epih.e2024055 (PMC11573489; doi:10.4178/epih.e2024055)
Supplement: Supplementary Material 2. — Duration of HIV diagnosis, initial ART, and death in the cohort data until 2017 [file epih-46-e2024055-Supplementary-2.docx]

Supplementary Materials 2. Duration of HIV diagnosis, initial ART, and death in the cohort data until 2017

|  | | Dataset 1 | Dataset 2 |
| --- | --- | --- | --- |
| The duration between HIV diagnosis to initial ART | | | |
|  |  | 1,336(100.0) | 1,978(100.0) |
|  | Within 3 months | 453(33.9) | 1,209(61.1) |
|  | 4 to 6 months | 100(7.5) | 124(6.3) |
|  | 7 to 12 months | 111(8.3) | 131(6.6) |
|  | 1 to 2 years | 150(11.2) | 123(6.2) |
|  | More than 2 years | 522(39.1) | 391(19.8) |
| The duration between HIV diagnosis to death | | | |
|  |  | 72(100.0) | 130(100.0) |
|  | Within 3 months | 7(9.7) | 48(36.9) |
|  | 4 to 6 months | 0(0.0) | 13(10.0) |
|  | 7 to 12 months | 2(2.8) | 11(8.5) |
|  | 1 to 2 years | 8(11.1) | 13(10.0) |
|  | More than 2 years | 55(76.4) | 45(34.6) |
| The duration between initial ART to death | | | |
|  |  | 60(100.0) | 89(100.0) |
|  | Within 3 months | 7(11.7) | 37(41.6) |
|  | 4 to 6 months | 3(5.0) | 9(10.1) |
|  | 7 to 12 months | 5(8.3) | 8(9.0) |
|  | 1 to 2 years | 11(18.3) | 7(7.9) |
|  | More than 2 years | 34(56.7) | 28(31.5) |
