# Supplementary material for: Comparison of HIV characteristics across 3 datasets: the Korea HIV/AIDS Cohort Study prospective, retrospective, and national reporting system
Source: Epidemiol Health. 2024 Jun 18;46:e2024055. doi: 10.4178/epih.e2024055 (PMC11573489; doi:10.4178/epih.e2024055)
Supplement: Supplementary Material 3. — Distribution of patients (n=number) according to intervals between diagnosis, starting ART, and all cause of death [file epih-46-e2024055-Supplementary-3.pptx]

## Slide 1
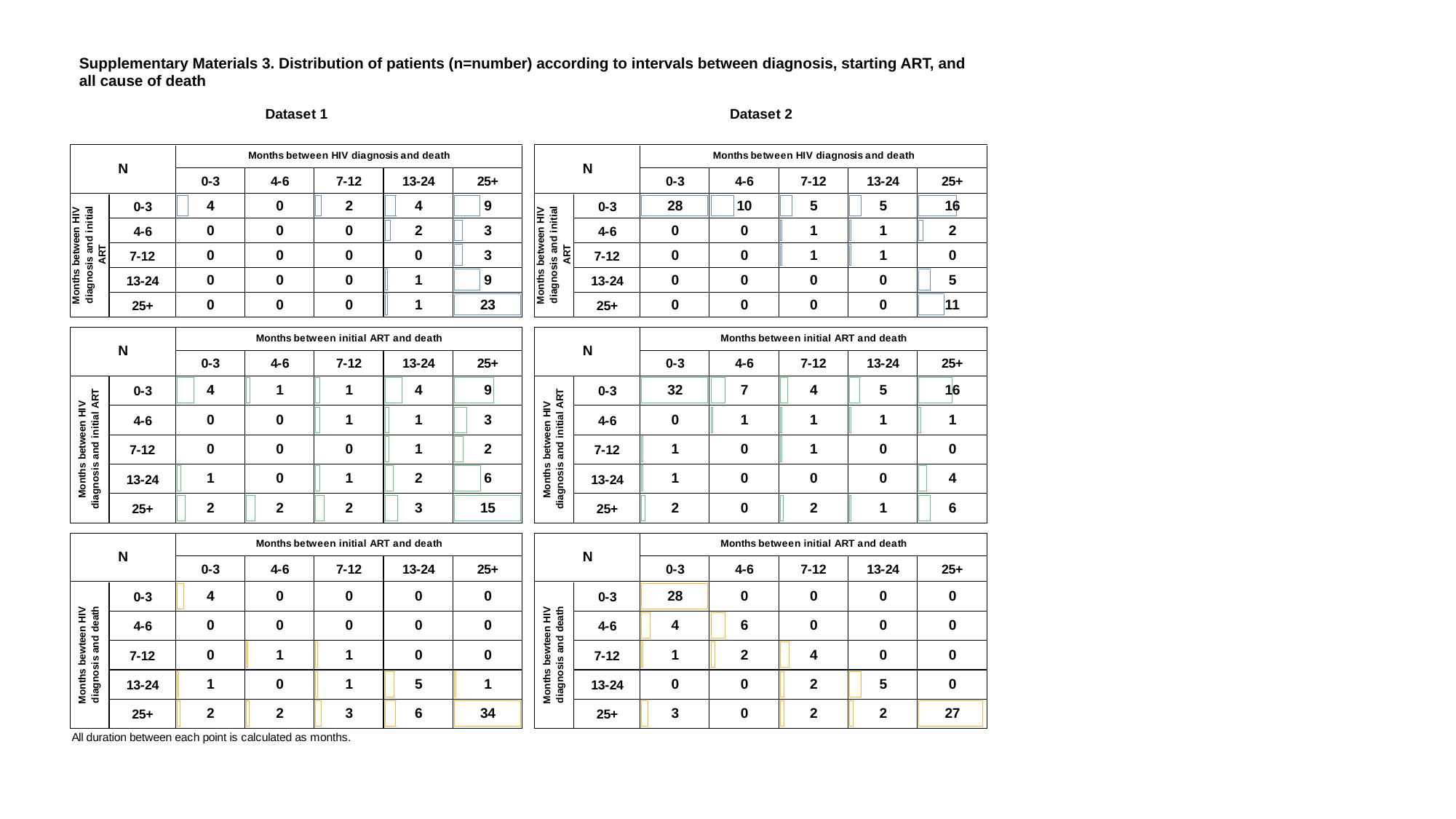

Supplementary Materials 3. Distribution of patients (n=number) according to intervals between diagnosis, starting ART, and all cause of death
