# Supplementary material for: Comparison of HIV characteristics across 3 datasets: the Korea HIV/AIDS Cohort Study prospective, retrospective, and national reporting system
Source: Epidemiol Health. 2024 Jun 18;46:e2024055. doi: 10.4178/epih.e2024055 (PMC11573489; doi:10.4178/epih.e2024055)
Supplement: Supplementary Material 4. — Causes of death by cohort enrollment until 2017 [file epih-46-e2024055-Supplementary-4.docx]

Supplementary Materials 4. Causes of death by cohort enrollment until 2017

|  | | Dataset 1^*^  (Antecedent / Immediate) | Dataset 2 |
| --- | --- | --- | --- |
| Cause of death | | | |
|  | AIDS | 15/0 | 50 |
|  | Opportunistic infection | 0/1 | 21 |
|  | Cancer | 2/1 | 20 |
|  | Heart / Brain / Vascular Disease and Dysfunction | 0/4 | 11 |
|  | Lung / Respiratory Disease and Dysfunction | 1/8 | 8 |
|  | Sepsis | 0/3 | 7 |
|  | Liver / Kidney Disease and Dysfunction | 3/3 | 2 |
|  | Multiple Organ Dysfunction / Failure | 0/2 | 2 |
|  | Others | 1/1 | 10 |
|  | Unknown | 0/52 | 29 |

^*^ Dataset 1 presents the underlying antecedent or immediate cause of death, and the values are not independent.
